# Smartphone-based alert of community first responders: A multinational survey to characterise contemporary systems

**DOI:** 10.1016/j.resplu.2025.100988

**Published:** 2025-05-21

**Authors:** Tore Marks, Bibiana Metelmann, Lorenzo Gamberini, Camilla Metelmann, Sebastian Schnaubelt, Federico Semeraro, Carolina Malta Hansen, European Resuscitation Guidelines 2025 Systems Saving Lives Writing Group

**Affiliations:** a Department of Anaesthesiology, University Medicine Greifswald, Germany; b Department of Anesthesia, Intensive Care and Prehospital Emergency, Ospedale Maggiore Carlo Alberto Pizzardi, Bologna, Emilia-Romagna, Italy; c Department of Anaesthesiology and Intensive Care Medicine, University Hospital Ulm, Germany; d PULS – Austrian Cardiac Arrest Awareness Association, Vienna, Austria; e Emergency Medical Service, Vienna, Austria; f Department of Emergency Medicine, University Medicine of Vienna, Austria; g Department of Anaesthesia and Intensive Care, Ospedale Maggiore Carlo Alberto Pizzardi, Azienda USL di Bologna, Italy; h Department of Clinical Medicine, University of Copenhagen, Denmark; i Copenhagen Emergency Medical Services, University of Copenhagen, Denmark; j Department of Cardiology, Herlev and Gentofte Hospital, University of Copenhagen, Denmark

**Keywords:** Out-of-hospital cardiac arrest, Cardiopulmonary resuscitation, First Responder, Qualification, Technology

## Abstract

**Aim:**

Several countries worldwide have implemented systems to alert community first responders (CFR) via smartphone applications to increase likelihood of survival after out-of-hospital cardiac arrest (OHCA). Substantial heterogeneity across CFR systems has been reported but recent reports are lacking. The European Resuscitation Council (ERC) conducted a survey to characterise and compare CFR systems focusing on requirements for joining CFR programs.

**Methods:**

An online survey with 28 questions regarding general system description, CFR qualification and training was conducted using SurveyMonkey between October 2024 and January 2025. The survey was shared via QR-code at the ERC Congress 2024, e-mail invitations to all ERC national resuscitation councils, the ERC Guidelines 2025 webpage, ERC social media, ERC newsletter, and personal e-mail invitations to research groups and CFR systems.

**Results:**

Thirty-five CFR systems from 19 countries participated in the survey. The majority of CFR systems (69%, *n* = 24) require some kind of Basic Life Support (BLS) training as a minimum qualification. In 80% (*n* = 28) the minimum age for participation is 18 years. App-specific training is offered by 51% (*n* = 18) and in 11% (*n* = 4) of CFR systems no dispatch centre is involved in the alert, 43% (*n* = 15) of systems alert exclusively to OHCA, and 17% (*n* = 6) of CFR systems only alert CFR to adult OHCAs.

**Conclusions:**

There are multiple CFR systems with a high degree of heterogeneity regarding minimum required CFR qualification and training as well as alerting modalities. Understanding these differences across systems is paramount to design studies to test the effect of CFR on patient outcomes.

## Introduction

In patients with out-of-hospital cardiac arrest (OHCA) the likelihood of survival decreases at least by 10% per minute without cardiopulmonary resuscitation (CPR).^1^, ^2^ Immediate bystander CPR and defibrillation can increase survival to >70%.^3^, ^4^ To facilitate prompt CPR and timely defibrillation, systems that alert community first responders (CFR) through smartphones have been established in several countries.^5^, ^6^, ^7^, ^8^, ^9^, ^10^, ^11^, ^12^, ^13^ CFR are individuals who have registered to be alerted to an emergency scene but have the option to decide whether or not to attend (e.g., volunteers notified via text-message or a smartphone application).^8^, ^14^, ^15^, ^16^ Current literature indicates that activating CFR alongside EMS increases bystander CPR^11^ and may decrease time to defibrillation, improve survival and neurological outcome.^7^, ^9^, ^17^, ^18^, ^19^, ^20^, ^21^, ^22^

CFR programs have been implemented following local protocols for recruitment and activation with great heterogeneity across systems.^12^ With a growing number of CFR programs, a comprehensive overview of contemporary CFR programs is lacking. To date, little is known about the requirements for registration with CFR programs such as Basic Life Support (BLS) proficiency level, prior training in BLS, as well as alerting modalities of CFR across systems. This information is crucial for better contextualising and comparing systems, as well as establishing common strategies for designing and activating CFR systems.

To gain insight into contemporary CFR programs, with a particular focus on requirements for CFR registration, the European Resuscitation Council (ERC) conducted a survey to compare existing CFR systems.

## Methods

### Questionnaire

A structured questionnaire was developed on the basis of published literature on CFR systems.^19^, ^23^, ^24^, ^25^ It was reviewed and revised by the members of the ERC Guidelines 2025 Systems Saving Lives Writing Group in a multi-step process (open discussion with the whole group, revision of the questionnaire, written individual feedback by the group, finalisation of questionnaire). The questionnaire was developed for the ERC Guidelines 2025 and designed to provide an update on existing CFR systems focusing on the required qualification and training of CFR. The final questionnaire consisted of 28 questions (single-choice, multiple-choice, open-phrased) in English. It began with a general description of the first responder system, including geographic characteristics, organisational information regarding the collaboration with the emergency dispatch centre, as well as indications and contraindications for alerting first responders. This section was followed by eight questions addressing the qualification of first responders, including any necessary certifications for registration. The next questions covered a potential app-specific training. The questionnaire concluded with two questions on on-duty non-healthcare personnel as first responders (see Supplement 2).

The Utstein Out-of-Hospital Cardiac Arrest Registry template was used as reference for terminology.^16^ First responders with a duty to attend (e.g., as in police or firefighter^26^) were not included.

### Distribution of the questionnaire

The online survey was performed using SurveyMonkey (SurveyMonkey Inc., San Mateo, CA, USA) from October 26^th^ 2024 to January 21^st^ 2025 using six routes:

(1) QR-code with a link to the survey at the ERC Resuscitation Congress 2024.
(2) E-mail-invitation from the ERC to all National Resuscitation Councils.
(3) Link to the survey on the ERC Guidelines 2025 webpage (October 26^th^ 2024, until January 21^st^ 2025).
(4) Link on the ERC social media (Facebook, Instagram, X, Threads, Bluesky) on November 11^th^ 2024.
(5) Link in the ERC Newsletter (December 5^th^ 2024), see Supplement 3.
(6) Individual invitations from the writing group to research groups and FR systems.

### Data analysis

Descriptive data analysis was conducted using Microsoft Excel Version 16.46 (Microsoft Cooperation, Redmond, WA, USA). Cartographic depiction was created using a “Natural Earth” map dataset^27^ and QGIS-LTR Version 3.34 (Open Source Geospatial Foundation, Delaware, USA). CFR systems were included if the question “Does your first responder system alert volunteer community responders?” was answered with “Yes”and key questions regarding the name of the app, qualification and training were reported. Duplicates were removed as follows: report of multiple responses per CFR scheme, providers were contacted for clarification; if unresolved, the most agreed-upon answer was chosen; for conflicting responses, “diverging answers” were noted; multiple valid answers or regional variations were combined; the most recent response was noted in case of multiple responses from the same IP address. CFR programs which could not be identified through an internet search and non-plausible answers (e.g. incomprehensible) were deleted. Results are presented as frequencies and proportions.

## Results

**Fig. 1:**
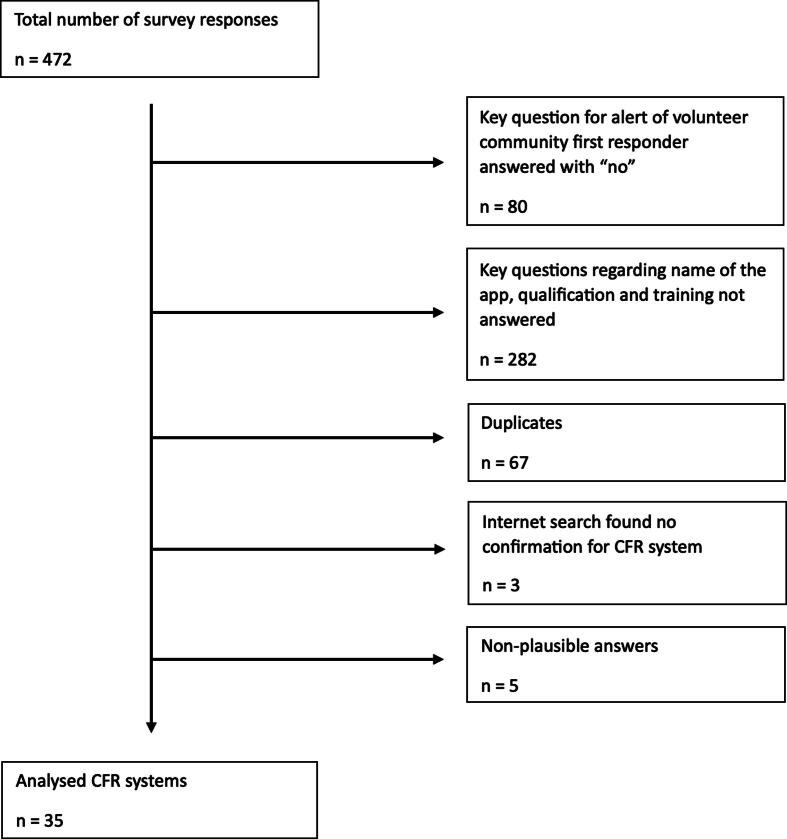
Flowchart for data reduction of survey responses.

A total of 472 respondents completed the questionnaire (Fig. 1) regarding 35 CFR systems in 19 countries (Table 1). The majority (*n* = 29) of CFR systems were in Europe (15 countries).

**Table 1:** Qualification, minimum CPR training and age limit for community first responders in systems worldwide.

Country	Operating System	Minimum Certification of CFR	Recertification of CFR	App specific training prior to or during registration*	Age limit for CFR
Australia	GoodSAM	None	Not applicable	None	18 years old
Austria	Team Österreich Lebensretter	BLS	No	None	18 years old
	Lebensretter.at	BLS	Yes	None	18 years old
Belgium	EVApp − Emergency Volunteer Application	BLS	Yes	Mandatory training	18 years old
Canada	FirstAED	BLS	Yes	Facultative training	18 years old
	GoodSAM	None, but BLS training offered	Not applicable	Mandatory training	21 years old
Denmark	Hjerteløber (HeartRunner)	None	Not applicable	Facultative training	18 years old
Finland	Virve	BLS	Yes	Mandatory training	18 years old
Germany	ASB SCHOCKT	Depends on local policy	Not applicable	Facultative training	18 years old
	Corhelper	BLS	Yes	Depends on local policy	18 years old
	KATRETTER	Depends on local policy	Not applicable	Depends on local policy	18 years old
	Land|Retter	BLS	No	Mandatory training	18 years old
	Mobile Retter	BLS	Depends on local policy	Depends on local policy	18 years old
	Region der Lebensretter (Region of Livesavers)	BLS	Yes	Facultative training	18 years old
Greece	iSAVElives	BLS	Yes	Diverging answers	12 years old
Ireland	NAS CFR App	BLS	Yes	None	18 years old
Italy	DAE RespondER	None	Not applicable	None	18 years old
	DAE FVG	None	Not applicable	None	18 years old
Luxemburg	112 CSU	BLS	Yes	Mandatory training	16 years old
The Netherlands	HartslagNu	BLS	Yes	None	18 years old
Poland	Pierwszy ratownik	None	Not applicable	Facultative training	18 years old
Romania	Există un erou în fiecare dintre voi	BLS	Yes	Mandatory training	16 years old
Singapore	myResponder App	None	Not applicable	None	No age limit
Sweden	Sms-livräddare	BLS	No	Facultative training	18 years old
Switzerland	ARMC	BLS	Yes	None	18 years old
	FirstResSO	BLS	Yes	None	18 years old
	FR Neuchâtel	BLS	Yes	Facultative training	No age limit
	FR Vaud	BLS	Yes	None	18 years old
	Momentum/Fondazione Ticino Cuore	BLS	Yes	Depends on local policy	18 years old
	Rescuetrack FirstResponder	BLS	Yes	Facultative training	18 years old
	Save a life	BLS	Yes	Facultative training	18 years old
United Kingdom of Great Britain	GoodSAM	BLS	Yes	Mandatory training (but only facultative for healthcare professionals)	18 years old
	NMA application	FROS 3	Yes	Mandatory training	18 years old
United States of America	PulsePoint	For deployment to private places: EMS certification or program-specific BLS training. For deployment to public places: none (depending on the region)	Depends on local policy	Depends on local policy	18 years old
	UnityPhilly	Special Training provided by the project	Not applicable	Mandatory training	18 years old

n.a. is “not answered”.

* “App-specific training” refers to all types of training, including legal training, training related to the app functions, as well as CPR training.

In 12 countries one active CFR system was reported and in 7 countries more than one active CFR system was reported (Supplement Fig. 1). A total of 6 active systems were reported from 4 countries outside of Europe: United States (*n* = 2), Canada (*n* = 2), Australia (*n* = 1) and Singapore (*n* = 1).

**Fig. 2:**
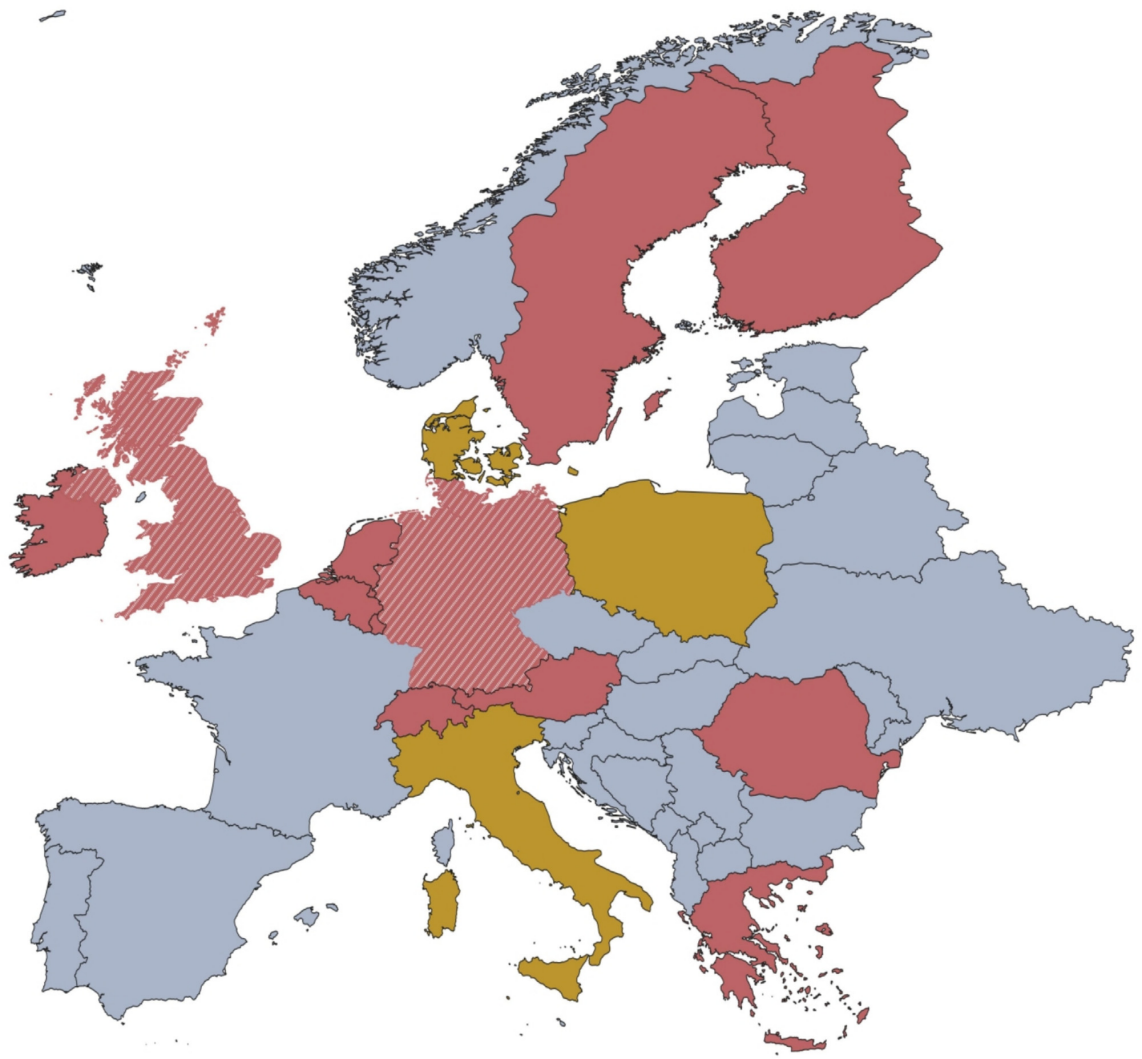
Minimum qualification requirements for joining European CFR systems; countries depicted in yellow: no qualification needed; countries depicted in red: BLS certificate as minimum level; countries depicted in striped red: multiple systems with different requirements; countries depicted in grey: no answer. As only a few systems outside of Europe completed the questionnaire, the graphical representation was limited to the region with the highest system density. (For interpretation of the references to colour in this figure legend, the reader is referred to the web version of this article.)

Fig. 2 shows a cartographic overview of active CFR systems in Europe and their qualification requirements.

The minimum qualification needed to register as a CFR differed within and across countries (Fig. 2). CFR systems in Denmark, Italy and Poland required no qualification, whereas systems in Austria, Belgium, Finland, Greece, Ireland, Luxemburg, The Netherlands, Romania, Sweden and Switzerland required a BLS certificate as a minimum level of certification. Among countries supporting more than one active CFR system, two reported deviating minimal CPR certification requirements: in Germany, CPR certification requirements ranged between none to qualification as healthcare professional. Systems in the United Kingdom required either BLS or FROS 3 (First Responder On Scene) as a minimum CPR certification.

In Singapore and Australia, no CPR certification was required. Systems in Canada required BLS certificates. In the United States, CPR certification requirements varied across systems.

### Qualification and training of community first responders

Table 1 provides an overview of CFR systems according to minimum CPR certification requirement, recertification, app specific training prior to or during registration, and minimum age for registration with CFR program.

Table 2 CFR presents systems according to activation modalities, alert criteria and exclusion criteria.

**Table 2:** Alerting modalities of community first responders in systems worldwide.

Country	Operating System	Activation only through emergency dispatch centre	Alert indication	Exclusion criteria for alert	Types of location for which the system is not alerted	Patient age limit as exclusion for alert
Australia	GoodSAM	Yes	Cardiac arrest	Crime scene*; Suicide	None	None
Austria	Team Österreich Lebensretter	Yes	Cardiac arrest	Crime scene*; highlevel roads (i.e. highways)	Care Homes; Medical Office; Other Medical Facilities	None
	Lebensretter.at	Yes	Cardiac arrest	Crime scene*; highlevel roads (i.e. highways); highly suspicious of traumatic cardiac arrest including fire	n.a.	18 years old
Belgium	EVApp − Emergency Volunteer Application	Yes	Cardiac arrest; Stroke; Anaphylaxis	Crime scene*; Suicide; Fire	None	18 years old
Canada	FirstAED	No	Cardiac arrest; Unconscious patient; Seizures Trauma; Overdose; Anaphylaxis;	Crime scene*; Suicide; Road Traffic Accidents; Fire; Limitation of Care; Palliative Care	Care Homes; Medical Office; Other Medical Facilities; Industrial Facilities	9 years old
	GoodSAM	Yes	Cardiac arrest	Crime scene*; Suicide; Trauma; Road Traffic Accidents; Fire; Limitation of Care; Palliative Care; Rescue of any kind (e.g. wilderness, water rescue)	Care Homes	None
Denmark	Hjerteløber (HeartRunner)	Yes	Cardiac arrest	Crime scene*; Suicide; Trauma; Road Traffic Accidents; Fire; Do not resuscitate orders Care; Palliative Care	Care Homes; Medical Office; Other Medical Facilities; Nursing homes; Rehabilitation centres	None
Finland	Virve	Yes	Cardiac arrest; Trauma; Stroke; Overdose; Anaphylaxis	Crime scene*; Suicide; Limitation of Care; Palliative Care	None	None
Germany	ASB SCHOCKT	Yes	Cardiac arrest	Depends on local policy	Depends on local policy	18 years old
	Corhelper	Yes	Cardiac arrest	Crime scene*; Suicide; Trauma; Road Traffic Accidents; Fire; Limitation of Care; Palliative Care; Depends on local policy)	Depends on local policy	Depends on local policy (e.g. only healthcare professionals to paediatric patients)
	KATRETTER	Yes	Cardiac arrest; Trauma; Stroke; Depends on local policy	Crime scene*; Suicide; Road Traffic Accidents; Fire; Electric injuries; Limitation of Care; Palliative Care; Depends on local policy	Care Homes; Medical Office; Other Medical Facilities (depends on local policy)	Neonatal (depends on local policy)
	Land|Retter	Yes	Cardiac arrest	Crime scene*; Suicide; Trauma; Road Traffic Accidents; Fire; Limitation of Care; Palliative Care	Medical Office; Other Medical Facilities	None
	Mobile Retter	Yes	Cardiac arrest; Unconscious patient; Stroke; Depends on local policy	Crime scene*; Suicide; Trauma; Road Traffic Accidents; Fire; Limitation of Care; Palliative Care (depends on local policy)	Care Homes; Medical Office; Other Medical Facilities; Prison; Asyl camp	Depends on local policy
	Region der Lebensretter (Region of Livesavers),	Yes	Cardiac arrest; Unconscious person	Crime scene*; Trauma; Road Traffic Accidents; Fire	Care Homes; Medical Office; Other Medical Facilities	None
Greece	iSAVElives	No	Cardiac arrest; Trauma; Stroke; Overdose; Anaphylaxis; “Anything that the person activating the alert feels they need help for”	Crime scene*; Suicide; Fire; Limitation of Care; Trauma	None	None
Ireland	NAS CFR App	Yes	Cardiac arrest, Trauma; Stroke; Anaphylaxis; Chest Pain;	Road Traffic Accidents; calls to major roads; Fire	None	None
Italy	DAE RespondER	Yes	Cardiac arrest	Crime scene*; Road Traffic Accidents; Fire; Limitation of Care; Palliative Care	None	None
	DAE FVG	Yes	Cardiac arrest	Crime scene*	None	None
Luxemburg	112 CSU	Yes	Cardiac arrest; Trauma; Stroke; Overdose; Anaphylaxis; Home birth	No exclusion criteria	None	n.a.
The Netherlands	HartslagNu	Yes	Cardiac arrest	Crime scene*; Suicide; Trauma; Road Traffic Accidents; Fire; Limitation of Care; Palliative Care; Unsafe situations; If BLS has been already started and an AED is already in use; (60+ exclusion criteria in total)	Care Homes; Medical Office; A&E, Traumacenter; Highway; Other Medical Facilities	1 year old
Poland	Pierwszy ratownik	No	Cardiac arrest; Trauma; Stroke; Anaphylaxis; Chest pain; Seizures	No exclusion criteria	All private places (e.g., homes, flats)	18 years old
Romania	Există un erou în fiecare dintre voi	Yes	Cardiac arrest; Trauma; Stroke, Overdose; Anaphylaxis; First aid in public spaces	No exclusion criteria	All private places (e.g., homes, flats); Military units	16 years old
Singapore	myResponder App	Yes	Cardiac arrest; Minor fire	No exclusion criteria	Other Medical Facilities	None
Sweden	Sms-livräddare	Yes	Cardiac arrest	Crime scene*; Suicide; Trauma; Road Traffic Accidents; Fire; Limitation of Care; Palliative Care; Ambulance assistance (depends on local policy)	Care Homes; Medical Office; Other Medical Facilities	18 years old
Switzerland	ARMC	Yes	Cardiac arrest; Unconscious patient; Heavy visible bleeding; Chest pain; Shortness of breath; Protection from heat or cold	Crime scene*; Trauma; Road Traffic Accidents; Fire	None	None
	FirstResSO	Yes	Cardiac arrest	Crime scene*; Suicide; Trauma; Road Traffic Accidents; Fire	Care Homes; Medical Office; Other Medical Facilities; Highways	None
	FR Neuchâtel	Yes	Cardiac arrest	Crime scene*; Suicide; Trauma; Road Traffic Accidents; Fire	None	18 years old
	FR Vaud	Yes	Cardiac arrest; Foreign Body Airway Obstruction	Crime scene*; Suicide; Trauma; Road Traffic Accidents; Fire; Limitation of Care; Palliative Care	Care Homes; Medical Office; Other Medical Facilities	None
	Momentum/Fondazione Ticino Cuore	Yes	Cardiac arrest; Trauma; Stroke; Overdose; Anaphylaxis; Home birth; Unconscious patient; all critical situations that cannot be reached by ambulance within 15–20 min	Crime scene*; Suicide; Trauma; Road Traffic Accidents; Fire; Palliative Care (depends on local policy)	Depends on local policy	Depends on local policy
	Rescuetrack FirstResponder	Yes	Cardiac arrest; Trauma; Electrical accident; Drowning accident	Crime scene*; depending on the situation	Depends on local policy	None
	Save a life	Yes	Cardiac arrest	n.a.	None	n.a.
United Kingdom of Great Britain	GoodSAM	Yes	Cardiac arrest; Trauma; Stroke; Overdose; Anaphylaxis	Crime scene*; Road Traffic Accidents; Fire	Medical Office; Other Medical Facilities	None
	NMA application	Yes	Cardiac arrest; Trauma; Stroke; Anaphylaxis	Road Traffic Accidents; Fire; Mental health	None	None
United States of America	PulsePoint	Yes	Cardiac arrest; insufficient breathing (depending on local policy)	Crime scene*; Suicide; Trauma; Road Traffic Accidents; Fire (depends on local policy)	All private places (e.g., homes, flats); Care Homes; Medical Office; Other Medical Facilities (depending on local policy and CFR qualification)	None
	UnityPhilly	No	Overdose	No exclusion criteria	None	None

n.a. is “not answered”.

* Crime scene was specified in the questionnaire as for instance suspicion of murder or shooting.

## Discussion

### Currently active systems

This study aimed to provide an overview of available CFR systems across the world, focusing on the minimum required age, qualification and CPR training for registering with current CFR programs, as well as alerting modalities.^12^, ^13^ Our main findings were that 35 CFR systems from 19 countries were reported, most of which (*n* = 29) were in European countries. The remaining systems were in North America, Singapore and Australia. We found great heterogeneity in terms of CPR certification requirements, conditions for which the systems were alerted (in addition to OHCA) as well as minimum age for registration. Understanding these differences across systems is paramount to interpret results and design studies to test the effect of CFR on patient outcomes.

Worldwide, five countries have more than one CFR system operating simultaneously. Even though several competing systems could promote technological progress, lack of compatibility between these apps could lead to situations, in which CFR registered in another system are not alerted although they are in close proximity to the OHCA patient. Greater harmonisation of systems – at least within countries, if not also in greater regions such as Europe – should be a future goal to facilitate implementation as well as monitoring system performance and improvement.

### Qualification and training

The majority of CFR systems required valid BLS training certification to register as a CFR. Frequent CPR training can significantly improve CPR quality and is also associated with psychological resilience in first responders.^28^, ^29^ Lay people have reported prior training to be beneficial when attempting resuscitation^30^ but increasing qualification criteria may reduce the available pool of CFR.^31^ Studies reporting system efficiency and patient outcomes according to CFR proficiency level are lacking and should be prioritised to guide CFR programs.

### Alerting modalities

With the exception of four systems, activation was carried out by the dispatch centre. A study highlighted the central role of dispatch centres in detecting and classifying an OHCA and activating CFR as they found that an automated alarm algorithm alerting CFR showed low accuracy in identifying OHCAs.^32^ In addition, lack of integration with the dispatch centre could lead to dispatching CFR to potentially dangerous situations.^33^ Only five systems have no exclusion criteria for alerting CFR. Dispatch centres can take on an essential gatekeeper function by filtering out potentially risky locations, such as crime scenes.

It is noteworthy that the survey was only completed by systems established in high-income (based on their Gross National Income) countries.^34^ A general transferability of the data to low- and middle-income countries is not possible as the current data supporting CFR programs are from integration with highly functional EMS systems. However, corresponding applications could help to improve medical care in less resourceful communities with longer ambulance response times. Our survey displays different approaches, some of which may serve as blueprints for low-resource settings. Nonetheless, ensuring thorough and high-quality training is essential to safeguard both the well-being of first responders and the patients they assist. Programs to empower local healthcare personnel aiming to improve care and outcomes in low- and middle-income communities such as the Acute Care Action Network^35^ could serve as inspiration to test CFR in less resourceful settings.

### Limitations

It was not possible to provide a precise response rate. However, most European countries were represented in the survey, thus representing most National Resuscitation Councils, which were the primary targets of the survey. Even though a broad range of stakeholders and researchers in resuscitation science were captured in the survey, there is a possibility that some CFR systems were not captured, especially outside of Europe.^34^ An additional literature and information search would have contributed to eliminate this bias, but publication bias for systems in low- and middle-income countries may exist. The survey was sent to the National Resuscitation Councils but not to EMS national or state directors of every European country. Since the survey was only distributed in English, a language bias cannot be ruled out. This might have led to an underrepresentation of non-English-speaking countries. Furthermore, the identity and effective involvement of the respondents in the CFR system could not be verified, potentially leading to imprecise answers. However, as described in the methods section, several strategies were implemented to ensure the accuracy of the data.

## Conclusions

A total of 35 CFR systems were reported in 19 countries across the world, most of which were in Europe, with considerable heterogeneity for minimum age and CPR qualification as well as criteria for system alert. This heterogeneity should be taken into account when evaluating and comparing CFR systems. Future research should seek to optimise CFR programs according to CFR training, population density, and EMS organisation as well as assess whether emerging technologies such as artificial intelligence or wearables may serve to optimise CFR systems.

## Consent for publication

Not applicable.

## Clinical trial number

Not applicable.

## Availability of data and materials

The datasets used and analysed during the current study are available from the corresponding author on reasonable request.

## Consent to participate

Informed consent was obtained from all individual participants included in the study.

## CRediT authorship contribution statement

**Tore Marks:** Writing – original draft, Validation, Formal analysis. **Bibiana Metelmann:** Writing – review & editing, Validation, Methodology, Investigation, Conceptualization. **Lorenzo Gamberini:** Writing – review & editing, Methodology, Investigation, Conceptualization. **Camilla Metelmann:** Writing – review & editing, Validation, Methodology, Investigation, Conceptualization. **Sebastian Schnaubelt:** Writing – review & editing, Validation, Methodology, Investigation, Conceptualization. **Federico Semeraro:** Writing – review & editing, Validation, Methodology, Investigation, Conceptualization. **Carolina Malta Hansen:** Writing – review & editing, Supervision, Methodology, Conceptualization.

## Ethics approval

This study was conducted in accordance with the ethical principles outlined in the Declaration of Helsinki.

## Funding

This research did not receive any specific grant from funding agencies in the public, commercial, or not-for-profit sectors.

## Declaration of competing interest

The authors declare the following financial interests/personal relationships which may be considered as potential competing interests: ‘LG is a member of the Scientific Committee of the Italian Resuscitation Council; SS is an ILCOR EIT Task Force member, ERC Advanced Life Support Science and Education Committee member, and Vice Chair of the Austrian Resuscitation Council; FS is the Chair of the European Resuscitation Council, an Emeritus member of the ILCOR BLS Working Group, and a member of the Italian Resuscitation Council Foundation. CMH reports receiving research grants from TrygFonden, Helsefonden, Novo Nordisk Foundation, Laerdal Foundation, Independent Research Fund Denmark, Capital Region of Denmark Research Fund. CMH is steering committee member for the RACE-CARS trial and the HeartRunner Trial, co-PI of the CARAMBA trial as well as a member of the ILCOR BLS Working Group. All other authors declare that they have no known competing financial interests or personal relationships that could have appeared to influence the work reported in this paper.’.

## Appendix A

**European Resuscitation Guidelines 2025 Systems Saving Lives Writing Groups:** Elena G. Bignami, Bernd W. Böttiger, Nino Fijačko, Koenraad G. Monsieurs, Hans van Schuppen.

## Appendix B Supplementary material

Supplement Fig. 1 Active community first responder systems in Europe; countries depicted in green: one system per country answered, countries depicted in striped green: more than one system per country; countries depicted in grey: no answer.

Supplement 2 Questionnaire.

Supplement 3 December issue of the ERC newsletter.
