# Supplementary figures and images for: Smartphone-based alert of community first responders: A multinational survey to characterise contemporary systems
Source: Resusc Plus. 2025 May 21;24:100988. doi: 10.1016/j.resplu.2025.100988 (PMC12167780; doi:10.1016/j.resplu.2025.100988)

**
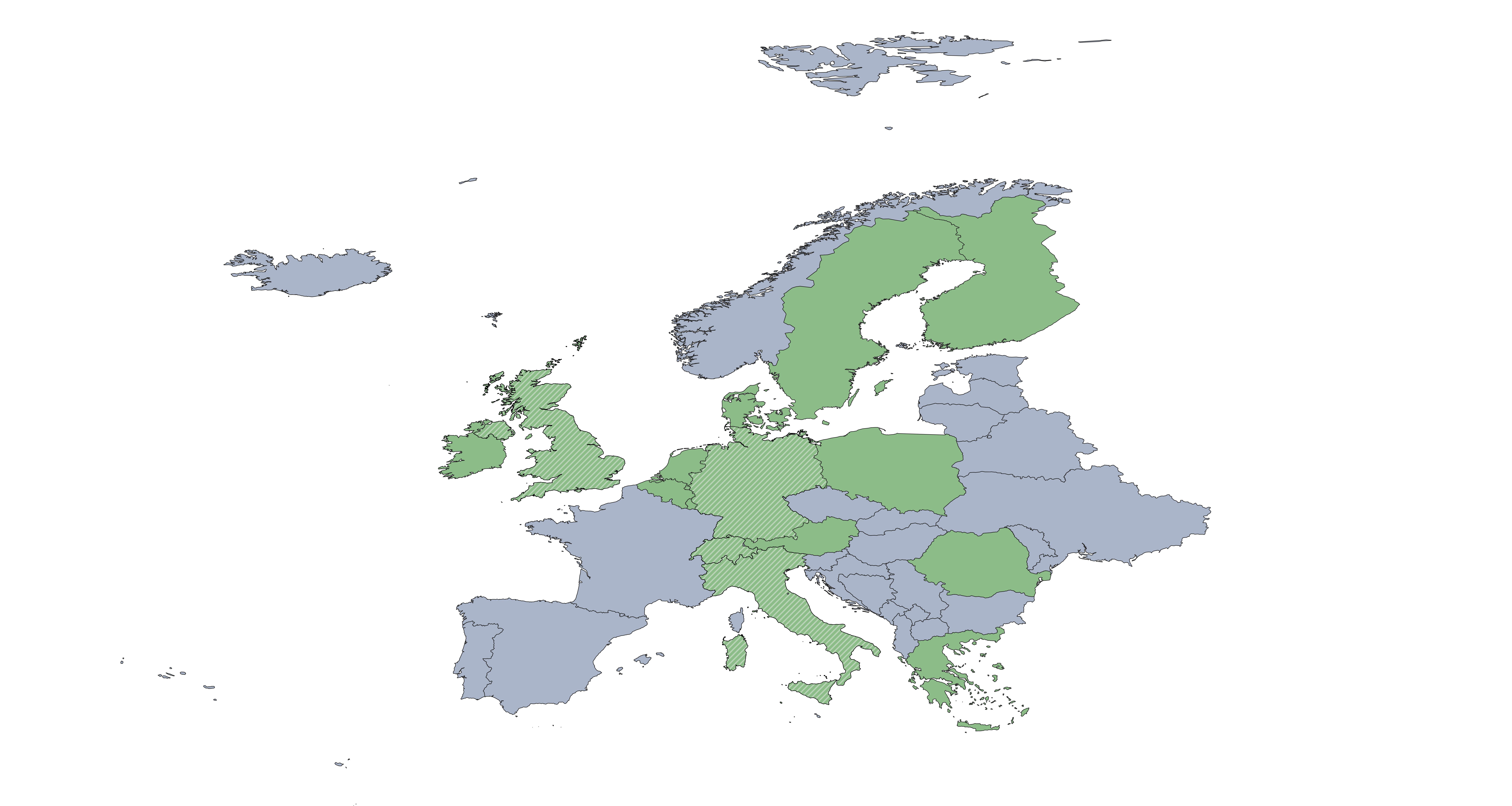
**

Supplement: Supplement Fig. 1 — Active community first responder systems in Europe; countries depicted in green: one system per country answered, countries depicted in striped green: more than one system per country; countries depicted in grey: no answer. [file mmc1.docx]
