# Supplementary material for: Smartphone-based alert of community first responders: A multinational survey to characterise contemporary systems
Source: Resusc Plus. 2025 May 21;24:100988. doi: 10.1016/j.resplu.2025.100988 (PMC12167780; doi:10.1016/j.resplu.2025.100988)
Supplement: Supplement 2 — Questionnaire. [file mmc2.pdf]

ERC first responder survey 2025

Question ID	Question	question type	Answer options
	**General questions**		
	In this survey, we are focusing on volunteer community responders. According to the Utstein 2024 definition, these are individuals who are alerted to an emergency scene but have the option to decide whether or not to attend (e.g., volunteers notified via a smartphone application). If you have different alert systems, we would like to ask you to fill out one questionaire for each system.		
1	Does your first responder system alert volunteer community responders?	Single choice	Yes
			No -> go to the second-last question (question 27)
2	What is the name of the app?	One line text	
3	In which region is the volunteer community responder system established?	One line text	
4	What is the catchment area (area covered) of your system? (Please indicate whether this number is in square km2 or square miles.)	One line text	
5	How many inhabitants live in the area covered?	One line text	
6	How many volunteer community responders are active in the system? If you don't know exactly give your best estimation.	One line text	
7	Are the volunteer community responders alerted through the emergency dispatch centre?	Single choice	Yes, activation only through dispatch centre.
			No, the volunteer community responders can be alerted without dispatch centre involvement.
8	How many volunteer community responders are sent to the scene (based they are available)? If the number depends on the situation, please state the maximum number as a limit.	Single choice	All those accepting the mission
			There is a limit --> how many?

9	Which indications trigger the alert of the volunteer community responders?	Multiple choice	Cardiac arrest
			Trauma
			Stroke
			Overdose
			Anaphylaxis
			Home birth
			Other (specify)
10	Are there any exclusion criteria for the alert of volunteer community responders?	Multiple choice	crime scene (e.g. suspicion of murder, shooting)
			suicide
			trauma
			road traffic accidents
			fire
			limitation of care
			palliative care
			Other (specify)
11	Are there any age limits as exclusion criteria for the alert of volunteer community responders?	Single choice	Yes (specify lower and upper limits)
			No
12	Are there any places the volunteer community responders are not alerted to?	Multiple choice	All private places (e.g., homes, flats)
			care homes
			medical office
			other medical facilities
			Other (specify)

			No, there are no places, where the volunteer community responders are not alerted to.
13	Are volunteer community responders alerted also at night time?	Single choice	Yes, all volunteer community responders are alerted
			Yes, only if the volunteer community responders want to
			No
	**Qualification**		
14	Which is the age limit for becoming a volunteer community responder in your system?	One line text	
15	Which kind of certification is needed to become a volunteer community responder in your system?	Multiple choice	No mandatory training or certification
			BLS training of any kind
			BLS training certified
			ILS training certified
			ALS training certified
			Healthcare professional
			Other (specify)
16	Does the certificate need to be valid (e.g. not expired) for becoming a volunteer community responder in your system?	Single choice	Yes
			No
			not applicable (no mandatory certification)
17	Is the volunteer community responder notified if his/her certificate expires?	Multiple choice	Yes - through the application

			Yes - by email
			Yes - by phone call
			No automatic notification
18	What happens if the volunteer community responder's certificate expires?	Single choice	Nothing happens
			He/She is deactivated until a new valid certificate is provided
			Other (specify)
19	Are volunteer community responders dispatched differently based on prior CPR training?	Single choice	Yes
			No
			Unknown
20	Are volunteer community responders instructed to perform pre-specified actions in an OHCA scenario?	Single choice	Yes - based on their skills (for systems recording volunteer community responders categories)
			Yes - based on their arrival order
			Yes - actions are chosen case wise by the Dispatch Centre
			No - actions are chosen case wise by the volunteer community responders themselves
21	What actions are the volunteer community responders expected to do?	Multiple choice	Perform CPR
			Bring AED to scene
			Administer drugs
			Airway opening (for acute airway obstruction not in cardiac arrest)
			Interaction with relatives

			Assisting EMS in transferring the patient to the ambulance
			Other (specify)
	**Training**		
22	Is there any app-specific training for volunteer community responders prior to or during registration?	Single choice	Yes, mandatory training
			Yes, facultative training
			No
23	If yes: What is the content of this app-specific training?	Multiple choice	CPR instructions
			skill training
			introduction into the technical system
			legal aspects (rights and duties)
			advice on how to deal with relatives
			contact adresses for psychological support
			Other (specify)
24	If yes: What type of app-specific training is offered?	Multiple choice	online training
			face-to-face training
			hybrid training
25	What equipment is provided to all volunteer community responders?	Multiple choice	Gloves
			Face shied
			Pocket Mask or similar

			bag valve mask
			First Aid kit
			AED
			Drugs
			Other (specify)
			None of the above
26	Can the Dispatch Centre provide assistance for volunteer community responders in performing CPR?	Multiple choice	Yes - all callers get telephone assisted CPR and this continues after the arrival of the first responder
			All callers get telephone assisted CPR, but this stops after the arrival of the first responder
			Yes - all callers get video assisted CPR and this continues after the arrival of the first responder
			All callers get video assisted CPR, but this stops after the arrival of the first responder
			If the first responders want to get assistance, they can contact the dispatch centre
			No assistance provided
			Other (specify)
27	Does your First Responder System alert **on-duty** non-healthcare personnel (e.g., typically fire and police)?	Single choice	If yes -> go to next question
			No --> end of questionnaire
28	Which categories of professionals can act as on-duty first responders in your system?	Multiple choice	taxi drivers
			couriers/riders
			firefighters
			policemen
			physio/-ergotherapists

			nurses
			doctors
			dentist
			Other (specify)
