# Supplementary material for: Smartphone-based alert of community first responders: A multinational survey to characterise contemporary systems
Source: Resusc Plus. 2025 May 21;24:100988. doi: 10.1016/j.resplu.2025.100988 (PMC12167780; doi:10.1016/j.resplu.2025.100988)
Supplement: Supplement 3 — December issue of the ERC newsletter. [file mmc3.pdf]

View this email in your browser

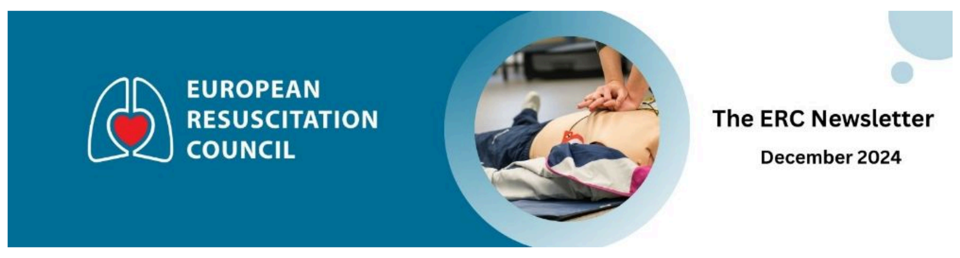

The ERC Newsletter is an informative publication providing regular updates on the latest developments and achievements in the field of resuscitation. It covers a wide range of topics, from scientific advancements to events and news highlights from the ERC and its affiliated organisations. The newsletter is a valuable resource for anyone interested in staying up-to-date with resuscitation science and practice. Whether you are a resuscitation science enthusiast, healthcare professional, or simply interested in our activities, the ERC Newsletter is a must-read.

Success Story: Resuscitation 2024

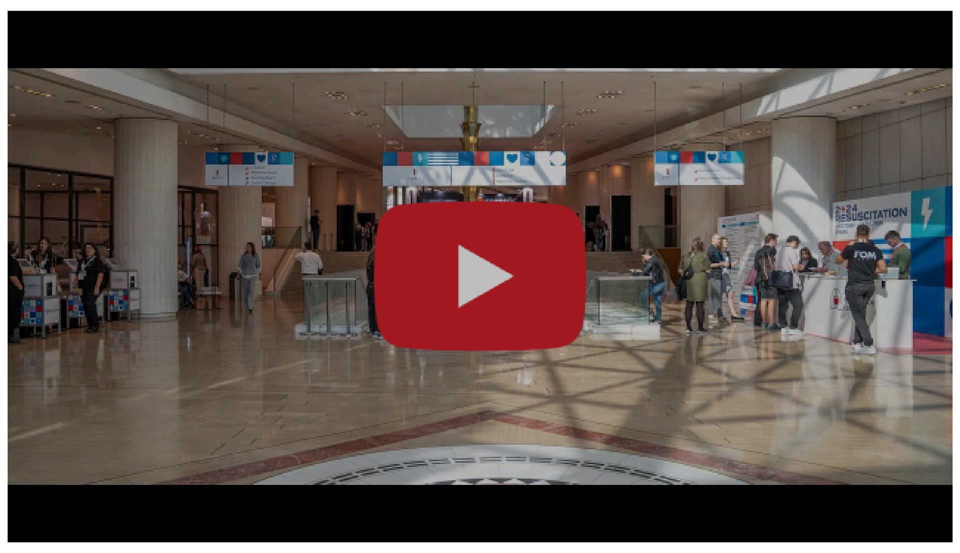

A moment to reflect on the success of the RESUS24 from Athens, Greece. The ERC Congress attracted around 1,400 participants from 69 countries, including 61 from lower and middle-income countries.

Subscribe to the ERC YouTube Channel

In the Spotlight

The Resuscitation Competition at the ERC Congress, 2024

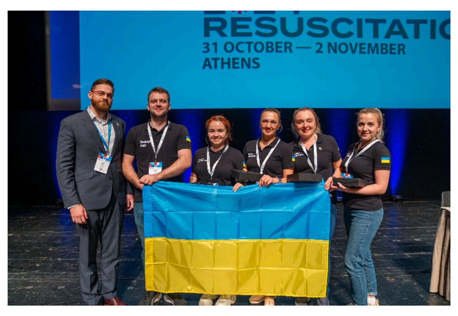

The Resuscitation Competition at the ERC Congress 2024 in Athens was an exhilarating display of skill and teamwork. This event gathered 14 top teams from around the world, highlighting their expertise in life-saving techniques under pressure. Participants took part in simulated scenarios that not only tested their resuscitation skills but also challenged their ability to collaborate and think critically in high-stakes situations. With a diverse range of teams competing, the atmosphere was electrifying as each group strived for excellence, driven by their passion for saving lives. Attendees had the opportunity to witness the dedication and talent of these teams in action, as they pushed the boundaries of resuscitation science.

We take pride in every participant of the Resuscitation Competition from each team. Their expertise and knowledge are unmatched, and their passion is truly inspiring. We were finally able to congratulate Team Ukraine as the 2024 winners.

NRC Meet at the ERC Congress, 2024

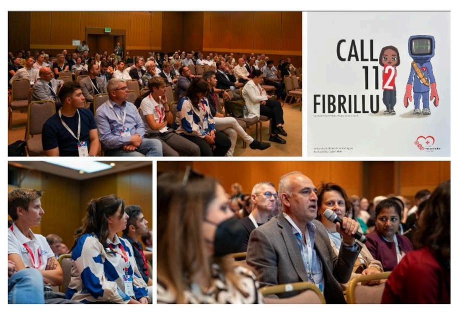

'(Re-)Connecting' was the keyword on the first evening of the ERC Congress, Resuscitation 2024. Around 100 people from 28 different national resuscitation councils (NRC) and ERC Board and Office members got together for the first NRC Meeting in a long time.

After the official welcoming by Şule Akın, the Representative of the NRCs in the ERC Board, the meeting kicked off with 'CALL 112 FIBRILLU' an illustrated booklet to teach school children about CPR. Tanya Esposito, Chair of the Malta Resuscitation Council (MRC), proudly presented the whole journey of this initiative as part of the long-standing efforts of the MRC to introduce CPR training in formal education. The publication was sponsored entirely by the Malta Resuscitation Council and is available in both printed and digital form. (https://www.resus.org.mt/post/112-fibrillu).

The next topic was translations of ERC training materials based on the ERC Guidelines. Marlie van Gils and Abel Roland from the ERC Office outlined the steps taken to ensure timely and high-quality translations. Aymeric Lejeune introduced 'NRC Days', set to launch in 2025, aimed at enhancing interaction and knowledge sharing between NRCs and the ERC while fostering alignment on future projects.

After the informative part, all attendees enjoyed drinks together, bringing back memories and creating new stories with others.

Science and Education: Applications invited for the Young ERC Editor

Resuscitation Plus, a companion title to the respected Resuscitation, is an open access international and interdisciplinary medical journal. The papers published deal with the aetiology, pathophysiology and prevention of cardiac arrest, resuscitation training and simulation, clinical resuscitation, and experimental resuscitation research. The journal aims to become the number one choice for authors wishing to submit methodically sound research relating to resuscitation in an open access journal. Further information about the journal is available in our editorial – Resuscitation plus – initial success and future direction. Aligned with the ERC and Young ERC visions – Resuscitation Plus is committed to equality, diversity and inclusion.

Resuscitation Plus is pleased to be officially recognised by the European Resuscitation Council and to work in partnership with the Young ERC to help develop the next generation of resuscitation scientists. We do this through participation in the Young ERC Resuscitation Science Masterclass Programme, running peer review training courses and supporting masterclass participants to take part in the peer review process with support from a mentor or editor and providing opportunities for publishing in the journal without charging an article processing fee.

In addition to the above activities, we also provide a fixed term (2 years) opportunity for an early career member of the ERC to serve as the Young ERC Editor for the journal. Kasper Lauridsen served as the inaugural Young ERC Editor. His term has now come to an end and we are therefore seeking the next Young ERC editor for Resuscitation Plus.

Role description
The Young ERC editor will be supported / mentored by the Editor in Chief and Associate Editors to participate in the full range of Editor tasks. This will include:

- Handling the peer review process through review of manuscripts, assignment of peer reviewers, providing editorial advice to authors, determining whether papers

- Participate in Editor- and Editorial Board meetings
- Conduct your activities with integrity and objectivity and with the policies of the journal and the publisher and COPE.
- Collaborate effectively with authors, reviewers, editors, editorial team and publisher
- Provide strategic input into the journal's development.
- Promote the journal to peers and colleagues.
- Collaborate with the Young ERC Committee and support their activities including the Young ERC Masterclass journal clubs, handling of Young ERC manuscripts and establishing Young ERC reviewers and editorial board members.

Time commitment
Serving as the Young ERC Editor is anticipated to require a commitment on average of 2-4 hours per week. This is typically spread over the week rather than occurring in concentrated blocks.

Remuneration
A small honorarium is provided to the Young ERC Editor

Essential criteria

- Higher degree related to health research (MSc or above)
- >3 publications in a peer reviewed journal
- Experience of critical appraisal gained through at least 2 years experience of undertaking peer review with reviewing tasks for at least 2 different journals
- Ability to work to deadlines and collaborate effectively within remote teams
- Free from commercial conflict of interest
- Member of ERC who is early in their resuscitation science career

Application process
Please complete the application form. This will be assessed by the journal editors and publisher and may be followed up by a short interview with the editors and members of the Young ERC Committee.

Closing date
15th December 2024

Submit the Application Form

Survey for the First Responders

The ERC has launched the survey for the First Responders.

In this survey, we are focusing on volunteer community responders. According to the Utstein 2024 definition, these are individuals who are alerted to an emergency scene but have the option to decide whether or not to attend (e.g., volunteers notified via a smartphone application). If you have multiple alert systems, we would like to ask you to fill out one questionnaire for each system.

By completing and returning this survey, you acknowledge that the data provided will be reported in aggregate form in a scientific publication. Rest assured that no personal data is collected.

This survey will take approximately 5 to 8 minutes. Kindly complete this survey by December 15.

We greatly appreciate your cooperation and look forward to your valuable input.

If you have any questions or comments regarding this survey, please contact guidelines@erc.edu.

Click to Fill Out the Survey

Young ERC at RESUS24: Open Meeting, Resuscitation Science Masterclass Forum, F.I.R.S.T. competition and poster sessions

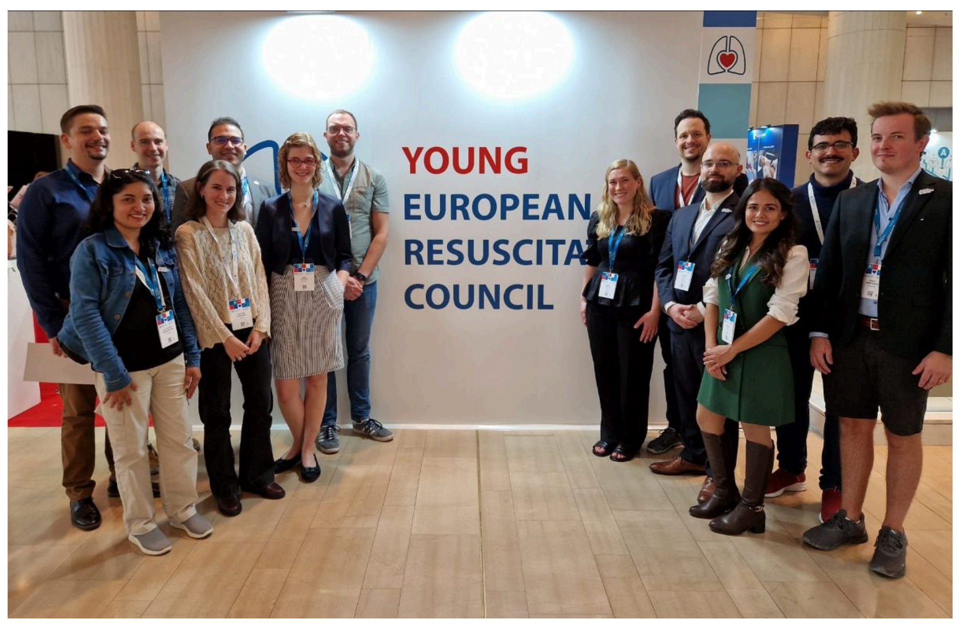

Young European Resuscitation Council at RESUS24 in Athens

At the ERC Congress in Athens, the Young ERC committee members had the pleasure of engaging peers and fellow members of the resuscitation community. The Young ERC committee supported the interactive congress atmosphere by facilitating the Resuscitation Olympic Games, coordinating inspiring early career sessions, and creating opportunities for early career visitors to make valuable connections. Many congress visitors stopped by the Young ERC booth for motivating discussions, meetings with friends, or networking with peers who share their passion for resuscitation.

Young ERC Open Meeting
On the first congress day, the Young ERC committee invited interested congress visitors to the Young ERC Open Meeting. More than 125 colleagues participated in the meeting, receiving a brief update on the Young ERC's work and an inspiring talk about mentorship in resuscitation science by Professor Vinay Nadkarni who has mentored over 150 clinician scientists.

Resuscitation Science Masterclass Forum
On the second day of the congress, the Resuscitation Science Masterclass Forum brought together past and present Masterclass participants, Masterclass faculty as well as many individuals with an interest in resuscitation science. Three former participants gave talks about the impactful opportunities that have emerged from their participation in the Masterclass, such as launching an important research project with international expert collaborators, gaining a new perspective on the paramedic career path by combining it with research activity and a new study program as well as moving from Norway to Italy to work on an exciting resuscitation research project. The Young ERC team is sincerely grateful for the interest in the Resuscitation Science Masterclass Forum and will continue to connect, inspire and support the next generation of the resuscitation community.

F.I.R.S.T. competition
The F.I.R.S.T. (Future Innovations in Resuscitation Science and Technology) competition was organized by the Young ERC and provided the audience with creative and innovative ideas on novel applications of artificial intelligence in resuscitation. The competition winners received one year of free ERC membership and were recognized during the closing ceremony. Thank you to all the contestants for sharing their innovative ideas and great presentations.

Poster moderations
This year's poster sessions provided many memorable and inspiring experiences. Hundreds of abstracts on novel and important resuscitation science projects were presented as posters during the congress. Together with senior faculty Young ERC committee members had the pleasure of moderating the poster sessions which led to many exciting discussions on current and upcoming resuscitation projects with the many brilliant resuscitation scientists. Thank you to all the researchers who presented their important scientific contributions in Athens!

Thank you for an amazing RESUS24 – We are already looking forward to seeing you again next year at RESUS25!

Trending on Social Media

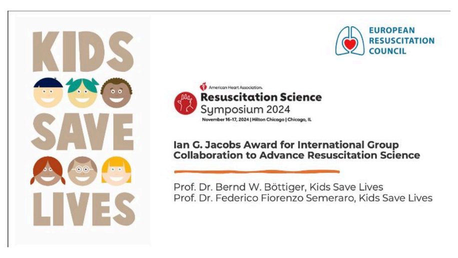

Exciting News for Kids Save Lives
View the post on LinkedIn

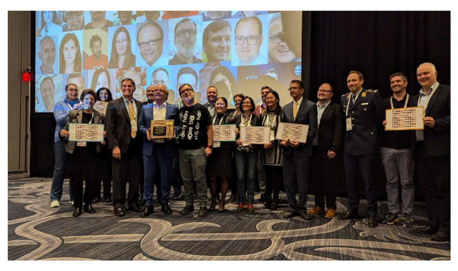

Kids Save Lives Makes History
View the post on Facebook

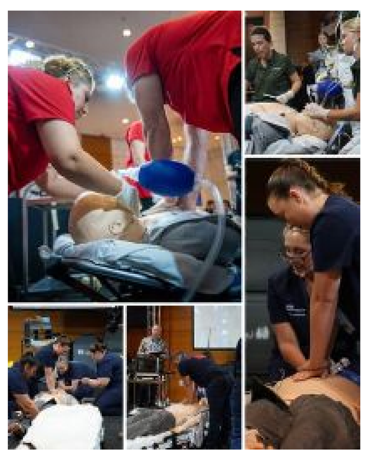

Sneak peek from the Resuscitation Competition
View the post on Facebook

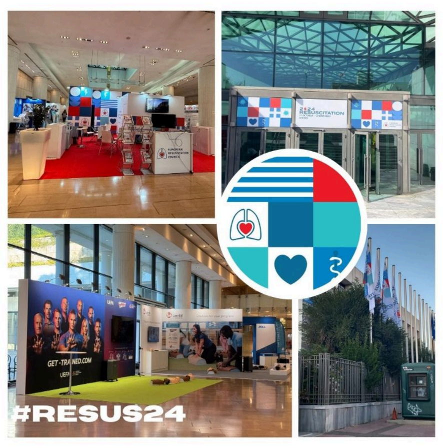

Athens Here We Go
View the post on LinkedIn

Join the ERC and Become a Member Today!

Share Share Share

Copyright © 2024 European Resuscitation Council, All rights reserved.

Want to change how you receive these emails?
You can update your preferences or unsubscribe from this list.

INTUIT mailchimp
